# Can Failed Back Surgery Syndrome Be Healed by Transverse Myelitis?

**DOI:** 10.7759/cureus.39680

**Published:** 2023-05-30

**Authors:** Elisa Moreira, Tiago Soares, Rafaela Evangelista, Ana Torres, Jorge Caldas

**Affiliations:** 1 Physical Medicine and Rehabilitation, Tondela-Viseu Hospital Center, Viseu, PRT

**Keywords:** longitudinally extensive transverse myelitis (letm), chronic pain, spinal injury, failed back surgery syndrome (fbss), chronic low back pain (clbp)

## Abstract

Failed back surgery syndrome (FBSS) is a condition characterized by persistent or recurring back pain following spinal surgery. Etiological factors for FBSS are being studied by investigators and clinicians in an attempt to organize them based on their temporal relation to the surgery event. However, many questions regarding the pathophysiology of FBSS remain, which has resulted in a lack of efficacy among its treatment options. In this report, we present a remarkable case of longitudinally extensive transverse myelitis (LETM) in a patient with a medical history of FBSS who was taking multiple pain medications but had persisting pain. The patient, a 56-year-old woman, presented with an incomplete motor injury (American Spinal Injury Association Impairment Scale D) and a neurological level of C4. Investigations revealed an idiopathic LETM that was unresponsive to high doses of corticosteroids. An inpatient rehabilitation program was initiated, resulting in favorable clinical progress. The patient no longer complained of back pain, and her pain medication was gradually discontinued. At the time of discharge, the patient was able to walk with a stick, dress and groom herself independently, and eat with an adapted fork without experiencing pain. As the pain mechanisms underlying FBSS are complex and not yet fully understood, this clinical case aims to contribute to the discussion of possible pathological mechanisms implicated in LETM that may have contributed to the shutdown of pain perception in a patient with a history of FBSS. By doing so, we hope to identify new and effective ways to treat FBSS.

## Introduction

Failed back surgery syndrome (FBSS) is defined by the International Association for the Study of Pain as a “lumbar spinal pain of unknown origin either persisting despite surgical intervention or appearing after surgical intervention for spinal pain originally in the same topographical location” [1]. Postoperative scar tissue can result in adhesions to the dura mater and damage nerve roots, and the release of inflammatory mediators can modify nerve-sensitive function, leading to chronic neuropathic pain [2,3]. Despite its debilitating effect on patients and relative prevalence among the population receiving back surgery, its treatment remains a challenge [1,4].

Longitudinally extensive transverse myelitis (LETM) is defined as spinal cord inflammatory lesions affecting at least three spinal cord segments and resulting in hyperintensities on sagittal T2-weighted MRI sequences [5]. Its clinical course is characterized by single or multiple attacks of para- or tetraparesis, sensory deficits, and bowel/bladder disturbances [6].

Here, we present a case of LETM in a patient with a medical history of FBSS, in which there was a dramatic change in the clinical course of FBSS.

## Case presentation

A 56-year-old Caucasian woman was admitted to the hospital with a two-week history of progressive bilateral weakness in her upper and lower extremities, which had left her bedridden. She also reported sensory changes in her right arm and dorsal region, as well as voiding difficulties. The patient denied any history of trauma or previous similar episodes. She had a medical history of FBSS and depressive syndrome, which resulted in a slight disability. Although she was unable to carry out all of her previous activities, she was still capable of independently managing her own affairs without requiring assistance (modified Rankin score 2). The patient was receiving follow-up care at a chronic pain unit for her uncontrolled pain. She was taking multiple medications, including vortioxetine 20 mg, clomipramine 25 mg, duloxetine 60 mg, clorazepate dipotassium 7.5 mg, diazepam 10 mg, pregabalin 200 mg, cyclobenzaprine 10 mg, and tramadol 100 mg daily. Additionally, she used acetaminophen and non-steroidal anti-inflammatory drugs during exacerbations. On neurological examination, she had an incomplete motor injury, American Spinal Injury Association Impairment Scale D, with a bilateral C4 sensory level and C5 motor level (she graded 4 in every key muscle except right C8 and T1 which graded 1), deep anal pressure but no voluntary anal contraction. Reflexes were increased in the upper and lower extremities with positive Babinski bilaterally.

**Figure 1:**
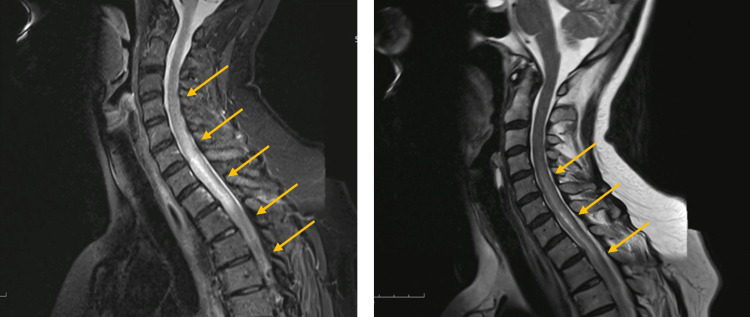
Cervical and thoracic spine MRI. MRI of the cervical and thoracic spine displaying a hyperintense signal on the short tau inversion recovery sequence on the left and gadolinium enhancement on the T2-weighted image on the right. These results suggest active inflammation in the spinal cord, which extends from the C4 to the D4 vertebrae (indicated by arrows) and are consistent with a diagnosis of longitudinally extensive transverse myelitis.

A gadolinium-enhanced MRI of the cervical and dorsal spine revealed an extensive hyperintense signal on T2 at the level of C4-D4, slight diffuse contrast enhancement, and slight dilation of the ependymal canal (Figure 1). The patient was diagnosed with LETM, and treatment with high doses of methylprednisolone was initiated for 12 days, with little or no improvement. Throughout the etiological investigation, a battery of examinations was conducted, ruling out central nervous system inflammatory demyelinating disorders, systemic inflammatory disorders, infectious causes, paraneoplastic syndromes, and deficiency syndromes. Consequently, an idiopathic LETM was diagnosed.

On the 13th day after admission to the hospital, she began inpatient rehabilitation and showed good progress in neurological and functional deficits. As she was no longer experiencing chronic pain, and her mood was stable, a gradual weaning off of pain and antidepressant medication was initiated under psychiatric supervision. We began with the suspension of tramadol, vortioxetine, and clomipramine to ensure a safe and comfortable transition, followed by the suspension of pregabalin. The patient continued to make good progress in her rehabilitation process and was capable, upon discharge, of walking with a stick, dressing and grooming herself independently, and eating with an adapted fork. Her biggest challenge was managing her neurogenic bladder with voiding problems, which required intermittent catheterization. At the time of discharge, she was free of pain and was transferred to a rehabilitation center to continue her rehabilitation program.

## Discussion

We present a case of FBSS with poorly controlled pain despite the implementation of analgesic treatment and regular follow-up in a chronic pain clinic, which resolved after LETM occurrence. The patient exhibited an incomplete spinal cord injury, likely presenting with a centromedullary syndrome, with greater involvement in the upper extremities and sensory deficits with hypoesthesia below the level of the lesion. Several mechanisms may be involved in pain inhibition following a spinal cord injury. Considering the pain neurotransmission mechanisms, phenomena such as central and peripheral pain inhibition, as well as cognitive or psychological factors, may be implicated.

Spinal cord injury leads, on its own, to the interruption of the transmission of painful inputs through the spinothalamic tract, which, as we know, is a sensory pathway that conveys nociceptive, temperature, crude touch, and pressure information from our skin to the somatosensory area of the thalamus [7]. Patients with FBSS, such as our patient, are often non-responders to analgesics, and minimally invasive approaches such as spinal cord stimulation (SCS) may be an effective therapeutic choice [8]. SCS consists of electrical stimulation of the dorsal columns of the spinal cord [8], and the proposed mechanism of how SCS works is believed to be more complex than just gate theory mechanics. It has been proposed that SCS-induced analgesia occurs not only via its effects on the spinal cord but via supraspinal components of the central nervous system as well as by inducing descending inhibitory pathways and inhibiting pain facilitation [9]. Similar to SCS, a spinal cord lesion can alter pain transmission and central perception, ultimately reducing or eliminating pain [10].

Proposed mechanisms for the development of chronic pain in FBSS include the irritation of nociceptive endings, axons, or processing circuits causing abnormal activity that is interpreted as pain [9,11]. It is known that sensory phenomena are a common complication of LETM [12], and that in most cases, inflammation may lead to sensitization of pain pathways, amplifying the perception of pain; however, in other cases, the spinal cord injury may affect pain transmission mechanisms, leading to a reduction or absence of pain.

Patients with FBSS typically have a long history of chronic pain [2]. Specific psychological factors that have been linked to poor outcomes in spinal surgery include high levels of depression, anxiety, poor coping, somatization, and hypochondriasis [2]. As pain persists, psychological and environmental factors become increasingly significant in disability, potentially exacerbating and perpetuating the pain [2]. In other words, these patients tend to focus heavily on their pain. In this case, we observe a shift in mindset from chronic pain to an acute inflammatory illness, with new challenges imposed beyond just pain.

One of the modalities for treating pain in FBSS involves therapeutic physical exercise, which has the additional benefit of teaching patients active coping mechanisms for pain, giving them a sense of control over their condition [2]. Strong evidence suggests that intensive interdisciplinary rehabilitation with functional restoration can improve function in FBSS management [2]. Applying these principles to the case presented, the early integration of the patient into an intensive and individualized rehabilitation program may have worked as a coping strategy, significantly contributing to her functional improvement and pain control.

To our knowledge, this is the first reported case in the literature that establishes a link between pain control in a patient with FBSS following LETM. Although it may seem overly simplistic to directly link these two occurrences based on a single clinical case, we consider this case to be a valuable opportunity to explore the physiopathology and pain mechanisms of FBSS and to contribute to the development of effective therapeutic strategies. We do not believe in coincidences, and we suspect that some relationship between the two conditions must exist. As FBSS management options are limited, investigating potential connections with other conditions such as LETM could shed light on new avenues for treatment.

## Conclusions

LETM is a serious and complex neurological condition that can affect various systems and functions of the body, including pain perception. It is important to note that the relationship between inflammatory spinal cord injury and chronic pain in FBSS is complex and dependent on various individual factors. Therefore, it is essential to conduct further research on pain mechanisms to better comprehend these conditions and develop effective pain management strategies. With a deeper understanding of the underlying mechanisms, healthcare professionals can provide more personalized and targeted care to patients suffering from FBSS and chronic pain.
